# Adherence to guidelines and suboptimal practice in term breech delivery with perinatal death- a population-based case-control study in Norway

**DOI:** 10.1186/s12884-019-2464-7

**Published:** 2019-09-09

**Authors:** Solveig Bjellmo, Sissel Hjelle, Lone Krebs, Elisabeth Magnussen, Torstein Vik

**Affiliations:** 1 Department of Obstetrics and Gynecology, More and Romsdal Hospital Trust, Postbox 1600, 6026, Aalesund, Norway; 2 Department of Clinical and Molecular Medicine, Norwegian University of Science and Technology (NTNU), Trondheim, Norway; 3 Department of Gynecology and Obstetrics, University of Copenhagen Holbaek Hospital, Holbaek, Denmark; 4 Department of Obstetrics and Gynecology, St Olav’s University Hospital, Trondheim, Norway

**Keywords:** Breech delivery, Mortality, Perinatal audit, Obstetrics

## Abstract

**Background:**

In a recent population-based study we reported excess risk of neonatal mortality associated with vaginal breech delivery. In this case-control study we examine whether deviations from Norwegian guidelines are more common in breech deliveries resulting in intrapartum or neonatal deaths than in breech deliveries where the offspring survives, and if these deaths are potentially avoidable.

**Material and methods:**

Case-control study completed as a perinatal audit including term breech deliveries of singleton without congenital anomalies in Norway from 1999 to 2015. Deliveries where the child died intrapartum or in the neonatal period were case deliveries. For each case, two control deliveries who survived were identified. All the included deliveries were reviewed by four obstetricians independently assessing if the deaths in the case group might have been avoided and if the management of the deviations from Norwegian guidelines were more common in case than in control deliveries.

**Results:**

Thirty-one case and 62 control deliveries were identified by the Medical Birth Registry of Norway. After exclusion of non-eligible deliveries, 22 case and 31 control deliveries were studied. Three case and two control deliveries were unplanned home deliveries, while all in-hospital deliveries were in line with national guidelines. Antenatal care and/or management of in-hospital deliveries was assessed as suboptimal in seven (37%) case and two (7%) control deliveries (*p* = 0.020). Three case deliveries were completed as planned caesarean delivery and 12 (75%) of the remaining 16 deaths were considered potentially avoidable had planned caesarean delivery been done. In seven of these 16 deliveries, death was associated with cord prolapse or difficult delivery of the head.

**Conclusion:**

All in-hospital breech deliveries were in line with Norwegian guidelines. Seven of twelve potentially avoidable deaths were associated with birth complications related to breech presentation. However, suboptimal care was more common in case than control deliveries. Further improvement of intrapartum care may be obtained through continuous rigorous training and feedback from repeated perinatal audits.

**Electronic supplementary material:**

The online version of this article (10.1186/s12884-019-2464-7) contains supplementary material, which is available to authorized users.

## Background

The optimal mode of delivery of a fetus in breech presentation has been debated for more than half a century. The essential question has centered around the risk for the fetus associated with vaginal delivery versus the risk for the pregnant woman associated with caesarean delivery (CD) in present and future pregnancies [1].

In 2000, the Term Breech Trial (TBT) was published [2]. This randomized controlled multi-center trial found lower perinatal mortality, neonatal mortality and serious morbidity in a group of women where CD was planned compared to a control group where vaginal delivery was planned. The study concluded that planned CD was better than planned vaginal birth for a fetus in breech presentation at term. The results of the TBT had a huge impact on clinical practice worldwide [2].

However, the study was also criticized [3–5]. In Norway, a group of national experts reviewed the evidence underlying these recommendations. Taking into consideration the much lower perinatal mortality in Norway than in the study populations included in the TBT, these experts concluded that vaginal delivery would still be safe, provided careful assessment of maternal and fetal status [6]. Nonetheless, clinical practice also changed in Norway where the vaginal breech delivery rate decreased from around 50 to 30% after 2000 [7].

In a recent study using prospectively recorded data from two national health registers, we found a significantly increased risk of intrapartum or neonatal mortality in singletons without congenital anomalies born vaginally in breech at term compared with singletons born vaginally in cephalic presentation [8]. However, limitations of register-based studies are the lack of detailed information regarding the delivery, as well as potential misclassifications for example due to congenital anomalies not identified at birth. A perinatal audit, defined as a systematic and critical analysis of the quality of the medical care of pregnant women and their newborns [9, 10] complement the findings provided by large register-based studies by identifying and analysing rare events, as well as provide a basis for proposing suggestions for improvement of care [11].

Therefore, as an expansion of our previous population-based study, we report the results of a case-control study completed as a perinatal audit of breech deliveries in Norway. The main aim was to explore if deviation from Norwegian guidelines was more common in breech deliveries associated with intrapartum or neonatal death than in breech deliveries where the infant survived. We also wanted to assess if death might have been prevented if the child had been delivered by planned CD, and whether suboptimal clinical management was more common in breech deliveries where the offspring died than deliveries where the offspring survived.

## Methods

In this population-based case-control study, all deliveries of children born in Norway during 1999–2015 in breech presentation at term (37–42 week), as singletons without congenital anomalies were eligible.

Case deliveries were defined as breech deliveries where the neonate died intrapartum or during the first 28 days after delivery regardless of mode of delivery. For each case delivery, two breech deliveries as close as possible in time, at the same hospital and by the same actual mode of delivery but who survived, were identified as controls.

Case and control deliveries were identified through the Medical Birth Registry of Norway (MBRN). This registry records demographic variables, as well as information on maternal health before and during pregnancy, interventions and complications during delivery and neonatal outcomes. Registration has been compulsory since 1967, ensuring prospective recording of the information at birth [12]. The unique 11-digit personal identification number for every Norwegian citizen made it possible to identify mothers of case and control deliveries. These mothers were mailed written information about the project and were given the opportunity to decline to be included in the study.

### “Open review”

Two of the authors, both consultants in obstetrics (SB and SH) reviewed the hospital records of all included deliveries. The available information included pre-pregnancy health, pregnancy related disorders, antenatal care, the course of the delivery, and the status of the offspring at birth and during the first 28 days of life. The two authors also identified when breech presentation was diagnosed. If the offspring died, available autopsy reports were reviewed. The two authors evaluated if the deliveries were in line with current Norwegian guidelines; for those who died, they also assessed if death might have been avoided if a planned CD had been performed. A structured questionnaire addressing the main points of the current Norwegian guidelines for breech deliveries **(**Short summary of the guidelines in Additional file 1) was developed by the first author (SB) and was completed by these two obstetricians. The newest revision of the Norwegian guidelines was published in 2014 [13]. The course of the actual delivery was evaluated against both the guidelines relevant at the time of delivery (‘historical’) and the current guidelines, and in addition, whether the delivery had been managed in accordance with best clinical practice (“optimal or suboptimal management”). The latter assessment was a subjective assessment by the two authors based on their clinical experience and the available written information. Thus, any conclusion of suboptimal management of a delivery does not imply malpractice. All assessments were completed independently by the two examiners followed by a conclusion in consensus.

### “Blinded review”

To reduce the risk of bias by knowing the outcome of the deliveries, two external examiners (EM and LK) reviewed the essential information of the course of all deliveries without being informed about whether the delivery was a case or control delivery. These two examiners are also experienced consultants in obstetrics working at other hospitals and in other cities than the unblinded authors, and they had both been involved in developing the national guidelines in their respective countries [14, 15]. For this part of the study, the first author provided a written detailed summary of the mothers’ pre-pregnancy health, pregnancy and delivery for all case and control deliveries. The summary included information on any pregnancy-related disorders, antenatal care, the results of ultrasound examination and pelvimetry, the partogram, the descriptions of surgical procedures as well as the gestational age and the birthweight of the child. Copies of electronic fetal heart monitoring, including cardiotocography (CTG) and ST waveform analysis (STAN) were also included. Information on Apgar scores and the status of the newborn was not provided. The two external examiners independently completed a questionnaire about the course of the delivery. In this questionnaire they were asked to indicate any deviations from the Norwegian guidelines [13], and whether they considered the management of the delivery to be suboptimal or not. They were also asked to predict whether they would expect the child to die or survive. Regardless of this prediction, they were also asked to indicate – for all case and control deliveries – whether, in case the child had died, death could have been prevented by planned CD.

In a consensus meeting, all four examiners agreed on potential avoidable deaths, if the deliveries were in line with the Norwegian guidelines, and if the management of the delivery was optimal or suboptimal.

### Statistical analyses

IBM SPSS statistics 25 was used for data analyses. Fisher’s exact test was used for bivariate comparison and *p*-values ≤0.05 were considered statistically significant.

## Results

**Fig. 1:**
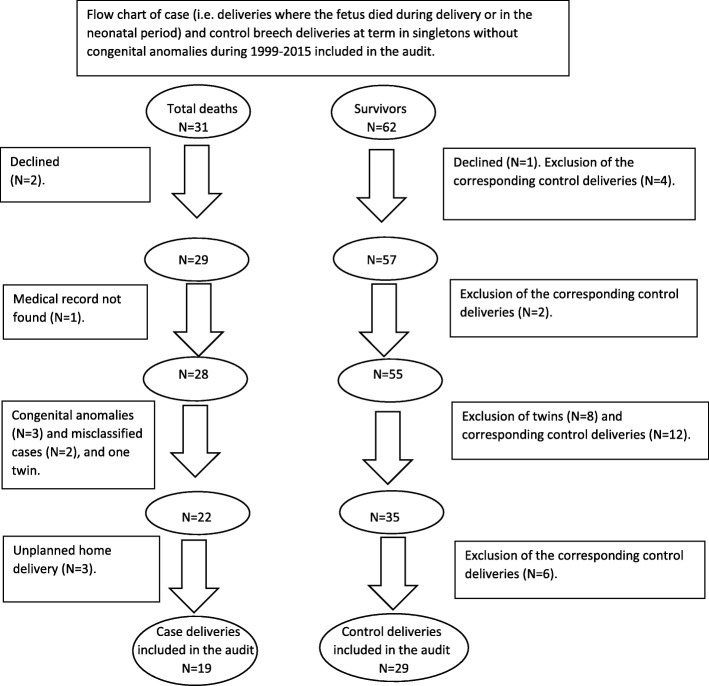
Flow chart of the study population

According to the MBRN, 31 term breech deliveries resulted in intrapartum death or death during the neonatal period of a child without congenital anomalies. The MBRN also identified 62 control deliveries. Two women in the case and one in the control group declined participation. Due to a misunderstanding, one delivery in the case and eight in the control group were twins, and these deliveries were excluded. A further case delivery was excluded because the hospital record could not be found. According to the MBRN, the child had a birthweight of less than 1500 g, and vaginal delivery was induced and completed at term suggesting that this delivery was most likely of a child with a congenital anomaly. Two case deliveries were classified as breech in the MBRN, while the review of the hospital records revealed that the two fetuses were in fact born in cephalic presentation. In three further case deliveries, the review of the hospital records revealed congenital anomalies that had not been recorded in the MBRN. Exclusion of the two case deliveries where the mothers declined, the one with lack of information, the twin delivery, the two with a fetus in cephalic presentation, and the three deliveries of a newborn with congenital anomalies resulted in 22 case deliveries available for further study (Fig. 1). Of these, three deliveries were home deliveries. In two of the home deliveries breech presentation had not been diagnosed before labour. The controls of the excluded case deliveries were also excluded from further study, as well as deliveries in the control group where the children were twins (*N* = 8). Thus, the case-control study included 19 case and 29 control in-hospital deliveries (Fig. 1).

The mean gestational age at delivery was 39.1 weeks (SD:1.2) in the case and 39.1 weeks (SD:1.3) in the control deliveries; the mean birthweight was 3369 g (SD: 475) in case and 3176 g (SD: 554) in control deliveries. Further background information is reported in Table 1, showing 12 (67%) boys among newborns of case deliveries compared with 14 (48%) boys in the control group (*p* = 0.312). Planned CD was done in a total of nine deliveries; three (16%) in the case group (all on maternal request) and six (21%) in the control group (three on maternal request; three because of fetopelvic disproportion). The three deaths following planned CD wereindependent of the delivery.

**Table 1:** Maternal and infants characteristics of case (infant died intrapartum or in the neonatal period) and control (infant survived) breech deliveries included in the study

	Case *N* = 19	(%) (100)	Control *N* = 29	(%) (100)
Maternal age				
> 17 years	2	([10)	0	(0)
22–34 years	14	(74)	23	(79)
> 35 year	3	(16)	6	(21)
Parity				
Nullipara	14	(74)	16	(55)
Multipara	5	(26)	13	(45)
Uterine scar				
Yes	0	(0)	3	(23)
No	5	(100)	10	(77)
Pre-pregnancy disorders^a^				
Yes	0	(0)	1^c^	(4)
No	16	(100)	25	(96)
Smoking during pregnancy^b^				
Yes	0	(0)	5	(21)
No	14	(100)	19	(79)
Mode of delivery				
Vaginal	10	(53)	14	(48)
Emergency caesarean	6	(31)	9	(31)
Planned caesaeran	3	(16)	6	(21)
The child:				
Sex				
Male	12	(67)	14	(48)
Female	7	(33)	15	(52)

^c^One mother who delivered vaginally had a congenital heart failure, but there was no contradiction against vaginal delivery

^a^Missing information in 3 cases and 3 controls

^b^Missing information in 5 cases and 5 controls

Among the remaining 16 case deliveries, ten (63%) were vaginal and six (37%) were emergency caesarean deliveries (Table 2). In four of these deliveries, death was considered independent of delivery, i.e. two uncomplicated vaginal deliveries and two emergency CD. One uncomplicated emergency CD was immediately following the diagnosis of breech presentation early in labour, while in the other emergency CD death occurred shortly after delivery and was assessed as mainly due to poor antenatal care with critical deterioration of fetal heart rate at admission before labour had started. Among the control deliveries, 14 (61%) were vaginal and nine (39%) were emergency CD.

**Table 2:** Characteristics of case and control in-hospital deliveries included in the study and where contractions and a vaginal delivery had started (i.e. those not performed as planned CD)

Vaginal or emergency caesarean delivery (ECD)	Case *N* = 16	(%) (100)	Control *N* = 23	(%) (100)
Undiagnosed before birth				
Yes	5	(31)	7	(30)
No	11	(69)	16	(70)
Mode of delivery				
Vaginal	10	(63)	14	(61)
ECD	6	(37)	9	(39)
Ultrasonography				
Yes	15	(94)	22	(96)
No	1^a^	(6)	1^a^	(4)
Type of breech (assessed with ultrasound)				
Frank breech	5	(31)	8	(35)
Complete breech	2	(13)	3	(13)
Footling	0	(0)	1	(4)
Not documented ^b^	9	(56)	11	(48)
Pelvimetry^c^				
Yes	10	(63)	11	(48)
No	6	(37)	12	(52)
Obstetrician present				
Yes	16	(100)	23	(100)
No	0	(0)	0	(0)
Pediatrician present				
Yes	14	(88)	14	(61)
No	2^d^	(12)	4^d^	(17)
Not documented	0	(0)	5	(22)
Fetal heart monitoring				
External	7	(44)	12	(52)
Internal	8	(50)	11	(48)
Not done	1^e^	(6)	0	(0)
Vaginal delivery	Case *N* = 10	(%) (100)	Control *N* = 14	(%) (100)
Anesthesia				
Epidural	8	(80)	11	(79)
No	2^f^	(20)	3^f^	(21)
Episiotomy (recommended in nullipara)				
Yes	8	(80)	10	(71)
No	2^g^	(20)	4^h^	(29)
Piper forceps				
Yes	7	(70)	5	(36)
No	3	(30)	9	(64)
Breech extraction				
Yes	2	(20)	1	(7)
No	8^i^	(80)	13^i^	(93)

^a^Undiagnosed breech discovered late in birth (at 9-10 cm) where the child was delivered shortly after

^b^Not documented in the medical record

^c^Pelvimetry is not prerequisite for vaginal delivery according to the guidelines

^d^According to current guidelines a pediatrician should be present at term breech vaginal delivery, but this was not required at the time of these six deliveries

^e^Cord prolapse at 10 cm

^f^Breech position diagnosed late in labour - born before epidural could be applied

^g^Both multiparas – episiotomy not a prerequisite in multiparas

^h^3 multiparas and one nullipara

^i^All births were assisted vaginal breech deliveries. (i.e. Løvset’s manoeuvre, Veit-Smellie-Mauriceau' manoeuvre or others)

The reasons for the emergency CD were fetal heart-rate abnormality (*N* = 6; three in each group), fetopelvic disproportion or failure to progress in labour (*N* = 4; all in the control group), footling breech presentation (one control), cord prolapse (two in the case group) and patient request at delivery (*N* = 2; one in each group).

Thus, of the 19 deaths in the case group, a total of seven (three planned CD, three independent of vaginal/emergency CD, and one due to poor antenatal care) were unrelated to the mode of delivery, while the deaths of 12 (eight vaginal deliveries and four emergency CD) would most likely have been prevented had a CD been planned and performed.

All four examiners concluded that all in-hospital case and control deliveries were in accordance with the guidelines recommended by the Norwegian Society of Gynecology and Obstetrics (Table 3).

**Table 3:** Deviations from current and actual Norwegian guidelines and suboptimal antenatal or intrapartum care for case and control term breech in-hospital deliveries in Norway 1999–2015, included in the study

	Case *N* = 19	(%) (100)	Control *N* = 29	(%) (100)	*p*-value^a^
Deviations from guidelines at the time of delivery					
Yes	0	(0)	0	(0)	–
No	19	(100)	29	(100)	
Deviations from current guidelines (2014)					
Yes^b^	2	(11)	4	(14)	1.0
No	17	(89)	25	(86)	
"Suboptimal care"^c^					
Yes	7	(37)	2	(7)	0.020
No	12	(63)	27	(93)	

^a^Fisher’s Exact Test

^b^According to current guidelines a paediatrician should be present at term breech vaginal delivery, but this was not required at the time of these six deliveries

^c^The assessment of suboptimal management is highly subjective and does not (necessary) imply malpractice

The four examiners concluded in consensus that the antenatal care or the clinical management of the delivery had been suboptimal in seven (37%) case deliveries. In six of these deliveries the examiners concluded that the response to fetal heart rate abnormalities had been inappropriate. The management of fetal heart rate abnormalities was also considered inappropriate in one control delivery, and the management of another control vaginal delivery was considered suboptimal, based on several risk factors for adverse outcome. Thus, suboptimal antenatal care and/or management of the delivery was more common among case than among control deliveries (*p* = 0.020; Table 3). Six of the nine deaths associated with suboptimal care occurred during the first half of the study period (all between 1999 and 2003), while three occurred during the second half (all three during 2012 and 2013).

Among the 12 potentially avoidable deaths, five occurred following a sentinel event despite appropriate care (cord prolapse in three cases; entrapment of the head in one and histopathological evidence of placental abruption in one case), three deaths were associated with suboptimal care, and in three cases the cause of death was attributed to a combination of a sentinel event (all difficult head deliveries) and suboptimal care. One of the 12 potential avoidable deaths occurred despite optimal care, with inconclusive cause of death.

## Discussion

In this study we found that all in-hospital case and control breech deliveries were managed in line with the guidelines recommended by the Norwegian Society of Gynecology and Obstetrics [13]. Planned CD was performed in three of the 19 case deliveries, and the death of these newborns was considered unrelated to delivery. Among the remaining 16 deliveries three out of four deaths might have been prevented if CD had been planned and performed. Finally, suboptimal management of the delivery was found to be more common in case than in control deliveries.

### Strengths and limitations

The strength of the study is the thorough review of the medical records, done independently by four obstetricians. A particular strength is that two examiners were not informed of the outcome when they reviewed and assessed the course of the deliveries.

The low number of deliveries is a limitation. Thus, lack of statistical significance between case and control deliveries must be interpreted with caution. Nonetheless, the percentages of most risk factors for adverse outcome of vaginal delivery were similar in the two groups. Moreover, statistically significant differences between the groups regarding suboptimal care or indicators of difficult deliveries (i.e. use of Pipers forceps and breech extraction) should also be interpreted with caution.

The review of the hospital records revealed that two case deliveries were misclassified as breech deliveries by the MBRN, while the children were in fact born in cephalic presentation. Correspondingly, children who were in fact born in breech may have been misclassified in the MBRN as being born in cephalic presentation. Thus, we may have missed one or more breech deliveries with suboptimal care or where the delivery was not in accordance with the Norwegian guidelines.

### Comparison with other studies

In a comparable study of term breech deliveries during 1982–92 in Denmark Krebs et al. studied the deliveries of 12 singletons without congenital anomalies who died intrapartum, or during the first week of life, and 23 controls. The authors reported that seven deaths (58%) were potentially avoidable [16] and that antenatal and/or intrapartum care was suboptimal in both in case (42%) and control (30%) deliveries. Regarding potentially avoidable deaths and deaths associated with suboptimal care, their results are in line with the findings of the present study. However, the proportion with suboptimal care in the control group was much lower in our (7%) than in the study in Denmark (30%). This difference may be explained by general overall improvements in both antenatal and intrapartum care between 1982 and 92 and 1999–2015.

Female fetuses are more likely than male fetuses to be in breech presentation at birth [17] and in line with this, there were more girls than boys in our control group. However, in the case group, two out of three infants were boys. Although not statistically significant, the differences in sex distribution between the case and control deliveries may be in line with studies showing that boys in general are more vulnerable than girls to fetal distress during labour [18, 19]. Another possible explanation for the predominance of boys in the case group is that boys have higher weight and head circumference than girls [20], which could explain why boys are more prone to complications during breech delivery. We are not aware that other studies have reported that the outcome of breech delivery may be different in boys and girls.

The proportion of fetuses in breech presentation undiagnosed before labour was slightly higher (30%) in our study than in other European studies (17–28%) [21, 22]. When breech presentation is discovered in labour it may be too late to evaluate the maternal pelvis and the fetal size, and external cephalic version may no longer be possible. However, studies published prior to the TBT did not find higher mortality or morbidity in deliveries of undiagnosed breech presentation, compared to those diagnosed before labour [23, 24]. Since it is likely that obstetricians at that time were more experienced in the management of vaginal breech deliveries, it may be a concern that as planned CD increases as the preferred mode of delivery, deliveries where breech presentation is diagnosed late in labour, may be managed suboptimally.

### Interpretation

Despite the fact that all deliveries were in line with Norwegian guidelines, 12 in-hospital deaths were potentially avoidable had CD been planned and performed. In eight of these cases, an unexpected complication occurred during delivery. Sudden, unexpected events also occur in deliveries in cephalic presentation and is a potential, albeit rare, complication of any vaginal delivery. However, in our population, seven of the eight sentinel events were events that occur more commonly (cord prolapse) or typically (difficult delivery of the head) for vaginal breech deliveries.

Intrapartum care was considered suboptimal in six of the potentially avoidable deaths. In all six cases, fetal heart rate had been misinterpreted. Suboptimal intrapartum care was more common in case than in control deliveries, and therefore the results of this audit may suggest that better training in the interpretation of fetal heart rate could have improved survival.

Although it was a post-hoc observation that six of nine deaths associated with suboptimal care occurred early in the study period, it may be noteworthy that in the year 2007 and 2008 some new therapeutic and diagnostic measures were introduced in Norway (i.e. therapeutic hypothermia and ST-analysis (STAN)). It may also be noteworthy that there has been a decrease in the occurrence of cerebral palsy between 1999 and 2010, probably due to overall improvement in antenatal, obstetric and neonatal care [25].

### Implication

Since all case deliveries were in line with current Norwegian guidelines the question may arise if these guidelines may be improved, or even completely replaced by a general recommendation to deliver all fetuses in breech by planned CD. Regarding the latter option, we have previously reported that the absolute risk for death associated with vaginal breech delivery is very low, and does not differ from the risk associated with planned CD, although it was slightly higher than for vaginal delivery in cephalic presentation [8]. The results of the present audit identifying several misclassified cases in the register suggest that the already very low absolute risk for death associated with vaginal breech delivery reported in the previous register-based study is probably even lower. Nonetheless, in line with the notion that “each baby counts” [26], the potential avoidable death of 12 newborns in our study is worrying. On the other hand, we found evidence of continuing improvement in the clinical management of vaginal breech deliveries, as the majority of deaths associated with suboptimal care occurred before 2003. There are also concerns regarding the future health of children born by CD, and regarding maternal and infant complications in subsequent pregnancies [1]. Based upon the results of our previous study, we estimate that to save one child, 500–600 mothers with a healthy baby presented in breech would need to have a CD [8]. As a comprehensive interpretation of the results of our previous population-based study, and the present audit, we therefore do not find it justified to recommend that current guidelines regarding vaginal breech delivery are replaced.

A secondary advantage of not replacing these guidelines is that obstetricians retain their skills in vaginal breech delivery which may benefit management of the deliveries of the 20–30% of fetuses in undiagnosed breech presentation and also of twin births with the first or second twin in breech presentation [27].

Regarding potential improvements of the guidelines, current Norwegian guidelines suggest that fetuses with an estimated weight between 4.0 and 4.5 kg may be suitable for vaginal delivery. This criterion is higher than the upper weight limit of 3.8–4.0 kg recommended in most other national guidelines [14, 28, 29]. In our study a stricter weight criterion for vaginal delivery might have prevented one death. However a recent study published in 2018 did not report increased infant morbidity after delivery of infants with a birthweight between 3.8 and 4.5 kg compared to those with a birthweight below 3.8 kg [30]. Another potential improvement of the guidelines might be to recommend pelvimetry in all cases of breech presentation before the decision of mode of delivery, since difficult delivery of the head was observed in four case deliveries. In the current Norwegian guidelines, pelvimetry may be done at the discretion of the responsible obstetrician. However, in two of these four difficult deliveries, pelvimetry had been done, in a further case, the mother was multipara (i.e. indicating appropriate pelvic size), and in the fourth case, pelvimetry could not be performed since breech presentation was diagnosed late in labour. Moreover, the evidence regarding whether pelvimetry may improve outcome of vaginal breech delivery is weak [31]. Thus, the present study did not identify issues that could lead to recommending changes in the current Norwegian guidelines. Vaginal delivery in upright position [32] was not observed in any of the deliveries included in this study. It is however, likely that delivery in upright position will be introduced as a recommended option for breech vaginal delivery in the next version of the Norwegian guidelines.

In 30% of all deliveries, breech presentation was not diagnosed before birth and one might speculate if ultrasound examination of the fetus late in pregnancy should be performed to confirm cephalic or breech presentation. Antenatal knowledge of fetal presentation might allow better planning of mode of delivery. In a recent study [33] including assessment of the cost effectiveness of universal ultrasound scanning near term of nulliparous women, the authors concluded that this examination would virtually eliminate undiagnosed intrapartum breech presentation. If the examination could be conducted by midwives using a portable ultrasound system, this would most likely be cost effective [33]. This would also make it possible to offer external cephalic version to more women eligible for this procedure [34–36]. However, consistent with earlier studies, the proportion of infants in undiagnosed breech presentation did not differ between case and control deliveries. Thus, it is unclear if the introduction of late US examination will improve survival taking into consideration the currently observed quality of breech deliveries in Norway.

In the assessment of optimal care, we considered six of the 12 potential avoidable deaths to be associated with suboptimal intrapartum care. In all of these cases, CTG was misinterpreted. Such misinterpretation occurs regardless of fetal presentation, and underscores the need for rigorous training of obstetricians in the assessment of pathological CTG. The authors of this study deem it necessary to interpret CTG in a consistent manner, regardless of fetal presentation.

In the discussion with the mother regarding the choice of mode of breech delivery it should be emphasized that unexpected acute complications may occur in vaginal breech delivery as well as in vaginal cephalic delivery, despite adherence to guidelines. However, even though some of these complications are more likely to occur in breech than in cephalic presentation they are extremely rare.

Finally, the review of the hospital records revealed that five of 31 case deliveries recorded in the MBRN as singletons without congenital anomalies in breech presentation were misclassified. An additional implication of the present study is therefore that, in line with a study by Goffinet et al. [37], results of large register-based studies need to be validated by in-depth studies.

Future research may address perinatal mortality and morbidity related to vaginal breech deliveries dependent on different guidelines and clinical handling in for example the Nordic countries or between other high-income countries.

## Conclusion

All in-hospital term breech deliveries included in this study were in line with the Norwegian guidelines for breech presentation. However, suboptimal care was more common in case deliveries than control deliveries. Among 12 potentially avoidable deaths, six were associated with suboptimal care. Further improvement of intrapartum care may be obtained through continuous rigorous training and feedback from repeated perinatal audits.

## Additional file

Additional file 1: Items recommended in the national Norwegian guidelines for breech presentation (2014). (ZIP 12 kb)

## Data Availability

Data may be made available upon reasonable request as deidentified participant data, provided extended approval by the the Regional Committee for Medical and Health Research Ethics in Central Norway and a Data Protection Impact Assessment.

## Abbreviations

CD: Caesarean delivery
CTG: Cardiotocography
MBRN: Medical Birth Registry of Norway
