# Supplementary material for: Adherence to guidelines and suboptimal practice in term breech delivery with perinatal death- a population-based case-control study in Norway
Source: BMC Pregnancy Childbirth. 2019 Sep 9;19:330. doi: 10.1186/s12884-019-2464-7 (PMC6734432; doi:10.1186/s12884-019-2464-7)
Supplement: Supplementary file 1 — Items recommended in the national Norwegian guidelines for breech presentation (2014). (ZIP 12 kb) [file 12884_2019_2464_MOESM1_ESM.zip › Supplementary_Guidelines_RevisedR1.docx]

| **Supplementary:** Items recommended in the national Norwegian guidelines for breech presentation (2014). |
| --- |
| - Ultrasonography ((estimating weight, and type of breech presentation (no footling presentation)). - Fetal weight estimated between 2000 g-4000 g (individual assessment between 4000-4500 g) - Pelvimetry (not obligatory). - Consider external cephalic version.   **Management of labor:**   - Staff skilled in breech delivery and immediate availability of facilities for safe emergency cesarean delivery (eg, anesthesia, obstetric, and pediatric personnel; surgical facilities and personnel). - Anesthesia (epidural or pudendal)*. - Oxytocin augmentation if needed. - Continuous electronic fetal heart-rate monitoring for fetal surveillance during labor. - Duration of active phase of second stage of labor should not exceed 60 min. - Episiotomy (Nullipara) - Assisted vaginal delivery of breech. |
| *****In the Norwegian guidelines, in contrast to many other guidelines, epidural anesthesia is recommended in breech deliveries. There are two reasons for this recommendation. First, Løvset’s manoeuvre and/or Pipers forceps are commonly and/or even routinely used in vaginal breech deliveries. These instrumental deliveries are best performed under anesthesia. Secondly, once epidural anesthesia is established, it can be used if vaginal delivery fails and an emergency CD is needed. |
